# Effectiveness of a community health worker-delivered care intervention for hypertension control in Uganda: study protocol for a stepped wedge, cluster randomized control trial

**DOI:** 10.1186/s13063-022-06403-9

**Published:** 2022-05-24

**Authors:** Rebecca Ingenhoff, Juliet Nandawula, Trishul Siddharthan, Isaac Ssekitoleko, Richard Munana, Benjamin E. Bodnar, Ivan Weswa, Bruce J. Kirenga, Gerald Mutungi, Markus van der Giet, Robert Kalyesubula, Felix Knauf

**Affiliations:** 1 Department of Nephrology and Medical Intensive Care, Charité - Universitätsmedizin Berlin, Charitéplatz 1, 10117 Berlin, Germany; 2 African Community Center for Social Sustainability, Nakaseke, Uganda; 3 Department of Pulmonary, Critical Care and Sleep Medicine, University of Miami, Coral Gables, USA; 4 MRC/UVRI and LSHTM Uganda Research Unit, Kampala, Uganda; 5 London School of Hygiene and Tropical Medicine, London, UK; 6 School of Public Health, Makerere University College of Health Sciences, Kampala, Uganda; 7 Department of Medicine, Johns Hopkins University School of Medicine, Baltimore, MD USA; 8 Makerere University Lung Institute, Kampala, Uganda; 9 Ministry of Health, Kampala, Uganda; 10 Department of Physiology, Uganda Department of Internal Medicine, Makerere University College of Health Sciences, Kampala, Uganda; 11 Department of Internal Medicine, Yale University School of Medicine, New Haven, CT USA

**Keywords:** Hypertension, Community health workers, Uncontrolled blood pressure, Cluster randomized control trial, Uganda

## Abstract

**Background:**

Over 80% of the morbidity and mortality related to non-communicable diseases (NCDs) occurs in low-income and middle-income countries (LMICs). Community health workers (CHWs) may improve disease control and medication adherence among patients with NCDs in LMICs, particularly in sub-Saharan African settings. In Uganda, and the majority of LMICs, management of uncontrolled hypertension remains limited in constrained health systems. Intervening at the primary care level, using CHWs to improve medical treatment outcomes has not been well studied. We aim to determine the effectiveness of a CHW-led intervention in blood pressure control among confirmed hypertensive patients and patient-related factors associated with uncontrolled hypertension.

**Methods:**

We will conduct a stepped-wedge cluster randomized controlled trial study of 869 adult patients with hypertension attending two NCD clinics to test the effectiveness, acceptability, and fidelity of a CHW-led intervention. The multi-component intervention will be centered on monthly household visits by trained CHWs for a period of 1 year, consisting of the following: (1) blood pressure and sugar monitoring, (2) BMI monitoring, (3) cardiovascular disease risk assessment, (4) using checklists to guide monitoring and referral to clinics, and (5) healthy lifestyle counseling and education. During home visits, CHWs will remind patients of follow-up visits. We will measure blood pressure at baseline and 3-monthly for the entire cohort. We will conduct individual-level mixed effects analyses of study data, adjusting for time and clustering by patient and community.

**Conclusion:**

The results of this study will inform community delivered HTN management across a range of LMIC settings.

**Trial registration:**

ClinicalTrials.govNCT05068505. Registered on October 6, 2021.

## Introduction

Non-communicable diseases (NCDs) represent a growing burden of morbidity and mortality, accounting for over 70% of the annual deaths worldwide. Over 80% of these deaths occur in low- and middle-income countries (LMICs) [1]. Hypertension is a serious medical condition and the major cause for premature death. The World Health Organization (WHO) estimates that globally 1.13 billion people suffer from hypertension, of which two thirds live in LMICs [2]. In 2015, on a global scale, one in four men and one in five women had hypertension. Fewer than one in five people suffering from hypertension have the disease under control [2]. Uncontrolled hypertension can result in significant morbidity including heart disease, stroke, vision changes, and hypertensive crisis, as well as premature death and increased costs of health care [2].

In Uganda, NCDs account for over 40% of the annual mortality [3]. In addition, more than a quarter of the Ugandan adult population has hypertension [4]. A study carried out in Uganda showed that only 9.4% of adults with hypertension had adequate blood pressure control and in urban and rural districts of Uganda over a fifth of persons aged 15 years and older suffer from uncontrolled hypertension [5, 6]. Hypertension control is crucial in reducing mortality and morbidity attributable to cardiovascular diseases. Furthermore, it is an important factor in lowering costs while using health care resources sustainably [7].

Inadequate control of blood pressure may be attributed to both provider-related and patient-related factors. Several studies imply that the primary patient-related factor is non-adherence with the prescribed antihypertensive medication. Therefore, the adoption of strategies that improve the long-term adherence to medication are recommended [8]. Among the factors which are associated with non-adherence are inadequate patient education towards disease management, high cost of medication and drug side effects. To overcome these obstacles in patient education and disease management, our study will test the effectiveness of a community health worker (CHW)-led intervention in rural Uganda.

CHWs are critical in bridging the gap in care between the community and the health centers. CHWs are usually community members who have completed a minimum of 7 years of education and are able to read and write in both English and Luganda. CHWs receive basic training on common health issues from the Ugandan Ministry of Health prior to being employed to the local communities where they support data collection and disease monitoring efforts. While working in a voluntary manner, they typically receive a small amount of monthly remuneration to support their work. They provide a link between the health sector and the communities [9]. CHWs are well utilized in the management of communicable diseases such as malaria, tuberculosis, and HIV [10, 11]. They have effectively provided a range of preventive and care interventions for maternal and child health and for infectious diseases in LMICs [12]. CHWs play an important role in ensuring that patients take their medications regularly and are additional a critical factor for improved patient survival [13]. A study in Uganda evaluated the care access and hypertension control in a CHW driven NCD program in rural Uganda. It indicates that hypertensive patients in a CHW program have a lower systolic blood pressure than the average systolic blood pressure of clinic patients [14]. Furthermore, a systematic review of sixteen trials that utilized CHWs for primary prevention and an early detection strategy in the management of NCDs in LMICs demonstrates that this strategy results in an increase in tobacco cessation and a decrease in systolic blood pressure, diastolic blood pressure, and blood sugar levels [15].

In Uganda, just like majority of LMICs, management of hypertension strains the already stretched health care delivery system. Evidence indicates that CHWs contribute positively to health care services as well as mortality and morbidity reduction. However, evidence on the role of CHWs in delivering interventions for NCDs on a primary care level is still developing and limited in Uganda and other LMICs [15, 16]. This lack of evidence is particularly the case for rural areas. Therefore, the purpose of this study is to evaluate the effectiveness of a community health worker-led intervention to control hypertension in rural Uganda.

## Design and methods

### Study setting

This study will be conducted in Nakaseke, Uganda, and coordinated by African Community Center for Social Sustainability (ACCESS). Since 2016, this research group along its international partners has established a network of CHWs trained in the identification and early diagnosis of NCDs such as hypertension, diabetes mellitus, chronic pulmonary obstructive disease (COPD), and kidney disease.

The study will be carried out in 102 villages within Nakaseke district. Study participants will be recruited from two NCD clinics within Nakaseke district: (i) Nakaseke hospital NCD clinic which is located in the general outpatient department of Nakaseke General Hospital and (ii) Life Care Center NCD clinic located at ACCESS which provide comprehensive NCD screening, care and management of patients. These clinics work closely with CHWs and are funded by Else Kröner-Fresenius-Stiftung and supported by Uganda’s Ministry of Health.

Three sub-counties have been selected for sampling of clusters. These include Nakaseke, Nakaseke Town Council, and Kasangombe. Sub-counties for sampling have been selected based on proximity to the two clinics as well as urban and rural representation of Nakaseke district. These sub-counties will be appropriate for implementation of the study due to excellent working relationships with the local communities through prior work; they do not currently have these kinds of interventions being implemented and have a rising number of NCD cases. Nakaseke sub country has an estimated total population of 21,500 people, with 4392 households, 6 parishes, and 41 villages, Nakaseke town council has an estimated total population of 9100 people with 1992 households; with 5 parishes and 17 villages and Kasangombe has an estimated total population of 26,600 people with 4851 households, with 4 parishes and 44 villages [17].

### Study population

The study population will include: participants with hypertension (systolic > 140 and/or diastolic > 90) attending the two NCD clinics, adults 18 years and above, participants residing in the three sub counties (Nakaseke Town Council, Nakaseke Sub County, and Kasangombe of Nakaseke district), and participants able to give informed consent. Patients diagnosed with hypertension but already controlled will be excluded. Pregnant women and patients with an expected life expectancy of less than one year will be excluded from participating in the study.

### Study design

**Fig. 1:**
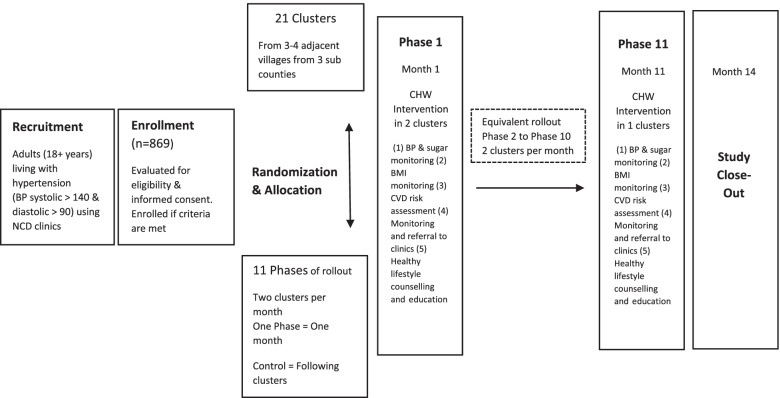
Recruitment design

**Fig. 2:**
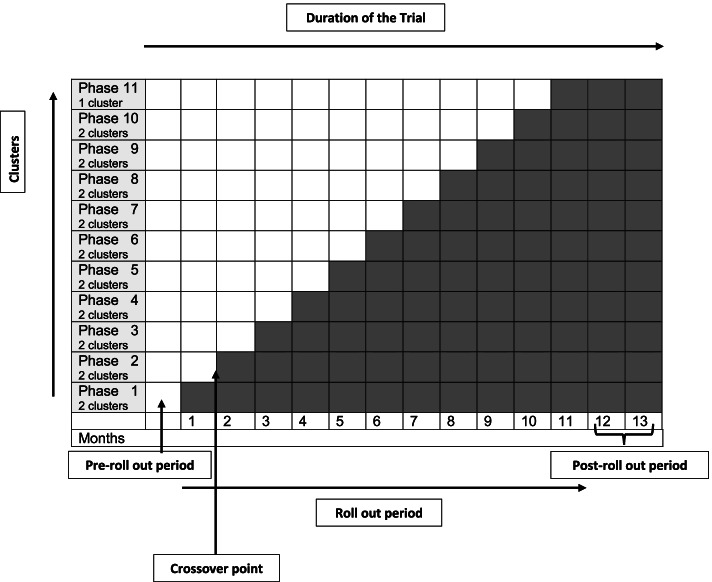
Schedule of enrollment and assessment of participants

The study will employ a closed cohort, superiority stepped wedge cluster randomized design. There will be a sequential crossover of clusters from the control to the intervention arms and the order of the cross over will be randomly determined (Fig. 1). This study will be conducted in 21 clusters within Nakaseke district. Each cluster will consist of 4–5 villages (Table 1). Participant recruitment will occur before randomization. At the beginning of the study period, we will collect baseline demographic data from each of the participants. All the villages within the study will eventually receive the intervention, thereby improving equity and acceptability. To minimize contamination, we will ensure that clusters are well separated to minimize social contact between participants in the intervention and control arms. Since all clusters will be observed under both the intervention and control arm, we anticipate that imbalances are less likely to occur. To further address the issue of contamination, individuals within the same cluster will be randomized to receive the intervention at the same time. Also, since the study will be conducted in a rural population, we expect adjacent neighborhoods to be far apart from each other, and this could additionally reduce the rates of contamination within the study area. In addition, CHW activities are directed to individuals at the level of the home and not large community groups or gatherings. This should decrease exposure of non-enrolled patients from outside randomized clusters to the interventions. We plan to rollout the intervention in two clusters per month and one cluster in the final month (Fig. 2).

**Table 1:** Formation of clusters within Nakaseke district

Sub county	No. of villages	No. of clusters
Nakaseke Town Council	17	4
Nakaseke Sub County	41	8
Kasangombe	44	9
Total	102	21

### Ethical considerations

The trial protocol was approved by the institutional review board (IRB) at the Makerere University College of Health Sciences School of Biomedical Sciences (SBS-2021-53) and the Uganda National Council of Science and Technology (HS1917ES). The trial was also registered with ClinicalTrials.gov (Identifier: NCT05068505) on October 6, 2021. Prior to any data collection, research staff will explain study purpose and procedures to participants. Written informed consent will be obtained for this and future ancillary studies, if applicable, from all the study participants prior to taking part in the study. In situations where a participant is unable to read or write, a thumbprint will be obtained, along with written signature from a witness. Access to identifiable individual-level data will be restricted to the study team. Protocol amendments will be approved by all regulatory parties prior to change in research activities. CHWs, research assistants, and the project team will also be trained in Good Clinical Practice and Protection of Human Research Participants.

### Training and quality control

The principal investigator (PI) and the project manager will conduct an all-study personnel-training session on the study protocol and Standard Operating Procedures before the initiation of the study. The research team will also be required to train in Good Clinical Practice, Human Subject Protection, and Responsible Conduct of Research. We will file copies of these research certificates in the investigation file. The PI will hold biweekly meetings with all the field staff and monthly meetings with all the investigators for their input and ensure that the study sticks to the study protocols and Standard Operating Procedures. The data will also be captured using tablet computers programmed with Research Electronic Data Capture (REDCap; Vanderbilt University, Nashville, USA). The study team will hold bi-monthly meetings to obtain continued support from the international partners from USA and Europe. The PI will also hold biweekly meeting to ensure that the study team sticks to the proposed study protocols.

### Community health worker training

A 5-day training will be held following the pre-established NCD curriculum for CHWs. Ministry of Health (MOH) village health team trainers will train CHWs in health promotion, basic research ethics, quality assurance, and control and use of tablet computers/redcap software. CHWs will also be trained in hypertension, screening risk factors, referral criteria, and need for community follow-up. Refresher training will be performed after 6 months.

### Logistic support

After the training, the CHWs will be equipped for their role with face masks, gloves, face shields, rain coats and gumboots, BP machines, weighing scales, tablet computers, glucometers, glucose strips, and urine dipsticks. They will also be remunerated with a monthly stipend.

### Recruitment, enrollment, and retention

Research assistants, who will obtain informed consent and collect the baseline and three-monthly data, will enroll study participants from the NCD clinics. Participants will be contacted by the CHWs, prior to the intervention rollout to schedule an initial home visit. The baseline assessment will be conducted following the WHO’s STEPwise approach to surveillance, a standard method for analyzing risk factors for NCDs, such as data collection on risk factors using the questionnaire and bio-physical measurements [18].

### Clustering and randomization

There will be a sequential crossover of clusters from the control to the intervention arms and the order of the cross over will be randomly determined. Participants will be recruited before randomization and the person responsible for the recruitment will be blinded from the allocations of interventions to the different clusters. The unit of randomization will be a cluster of 3-4 adjacent villages. A list of all villages from the three sub counties is available, and a total of 21 clusters will be designed from the three sub counties (Table 1). Clusters will be randomly assigned to the eleven phases of intervention rollout. Details of the timing of cluster allocation and randomization will be maintained in a secure document available to the project manager and PI and unavailable to those who enroll. The investigators and the participants will not know what groups will be randomized before allocation of the intervention. Because it is a CHW-led intervention, study investigators and participants cannot be concealed after enrolling. The allocation sequence will be randomly generated using a computer by our statistician and will be provided to the project manager at the time of enrollment of a new cluster.

### Primary outcome

Decrease in the average systolic pressure between the intervention and control arms.

### Secondary outcomes

Proportion of participants with blood pressure control, defined as blood pressure < 140/90. Glycemic control defined as a reduction > 1 in HbA1C. Composite negative clinical CVD outcomes including CVD related admissions, stroke, myocardial infarction, and incident heart failure will also be assessed as secondary variables. We will additionally measure quality of life through the EuroQol-5D-5L [19]. We will measure effectiveness measures including adoption and fidelity to the intervention over time.

### Independent variables

• Sociodemographic characteristics of the participants such as age, sex, education level, and marriage status

• Life style characteristic on: diet, tobacco use, and alcohol consumption

• Medical history including family history on NCDs such as diabetes, hypertension, and other co-morbidities like HIV and CKD

### Sample size determination

The primary outcome will be a change in systolic blood pressure between the intervention and control group. To compute the required sample size for the step wedge design, we first computed the required sample size if we were to implement an individual randomized trial. By conservative estimates, we estimate a 6-mmHg difference in systolic blood pressure between the intervention and control group [20, 21]. Accounting for the stepped-wedge design and constant cluster sizes (40) within the sample, with a fixed number of clusters (*n* = 21) and a power of 90%, at 0.05% level of significance, a sample size of 869 will power the study to detect an absolute minimally important clinical difference (MCID) of 6 mmHg in the mean systolic blood pressure between the intervention and control arms. We intend to roll out the intervention within two clusters each month and within one cluster during the final month.

### Sampling frame

A complete list of confirmed cases of hypertension attending the two NCD clinics and residing in either Nakaseke Town Council, Nakeseke Subcounty, or Kasangombe generated by the data manager.

### Intervention

The multi-component intervention will be centered on monthly pro-active household visits by trained CHWs for a period of 1 year.

It will consist of the following:

- Blood pressure and sugar monitoring. CHWs will be trained in measuring blood pressure using a digital blood pressure machine
- BMI monitoring
- Cardiovascular disease risk assessment
- Use of checklists to guide monitoring and referral to the clinics. Checklists will be filled in and handed over to the project staff for review at the end of every week
- Healthy lifestyle counseling and education. CHWs will be trained in a curriculum regarding home health education and strategies regarding behavioral change communication. The education will be provided to participants and all the family members during the home visit
- Follow-up during home visits to ensure that patients received all prescribed medications and to encourage them to adhere to them
- Providing patient-education using PocketDoktor™ booklets, an established education tool among hypertensive patients in rural settings [22]

During home visits, CHWs will remind patients of follow-up visits and encourage clinic attendance.

### Usual care

This involves monthly to three monthly patient visits to any of the two existing NCD clinics in Nakaseke. Clinics use the adopted tools for implementing WHO PEN (Package of Essential Non-Communicable Diseases Interventions) in the management of patients [23]. Routine clinic activities include taking of vital signs, group counseling, and review by the clinicians, medication prescription, and medical investigations as well as drug prescription and administration. All the participants will continue with the usual care. Implementing the CHW approach will not require alteration to usual pathways including use of any medication, for both trial arms. There are no restrictions regarding concomitant care during trial.

### Discontinuation

Participants who chose to discontinue from the intervention or the protocol will not have individually data collected through the fieldworkers. Analysis will be done through intention to treat.

### Procedures

Research assistants will collect sociodemographic data, exposure history and co-morbidities.

i. An average of three blood pressure measurements taken in a seating position after 10 minutes of rest according to recent guidelines [24] using an automatic Omron M4-1 sphygmomanometer. The blood pressure will be classified according to National Institute of Health (NIH) guidelines

ii. Height using a portable stadiometer to the nearest decimal point in centimeters

iii. Weight with digital Seca mechanical scales to the nearest kilogram. We will calculate for the BMI from the formula BMI = (𝑊𝑒𝑖𝑔ℎ𝑡 in 𝑘𝑔) ÷ (𝐻𝑒𝑖𝑔ℎ𝑡 in 𝑚) Using the NIH guidelines, the BMI will be classified as underweight (< 18.5), normal (18.5–24.9), overweight (25.0–29.9), and obesity (30.0–34.9) [25]

iv. Waist circumference and hip circumference will be obtained using a tape measure to the nearest decimal point in cm. This will be used to obtain the waist to hip ratio

v. Random and fasting blood sugars (RBS/FBS) will be measured using capillary blood

### Data collection and management

We will collect the study data on secure password-protected tablet computers with Research Electronic Data Capture (REDCap; Vanderbilt University, Nashville, USA). The data manager will crosscheck for completeness and correctness. It will then be exported to STATA 15 for analysis. We will conduct monthly checks of the data to assess completeness and outliers by the data manager and PI.

### Data analysis

Patient characteristics will be summarized using proportions for categorical variables. Means (standard deviations) and medians (interquartile ranges) will be used for continuous variables by exposure period (intervention and control periods). The chi-square and Fisher’s exact test will be used to test for differences in participant characteristics for categorical variables by exposure period status. The one-way ANOVA will be used to compare means between the two periods for normally distributed continuous variables, and Kruskal-Wallis test will be used for non-normally distributed continuous variables.

We will follow the intention-to-treat principle, and clusters will be analyzed according to their randomized crossover time irrespective of whether crossover was achieved at the desired time. Mixed effects logistic regression models will be used to assess the impact of the intervention on the primary outcome. These models will be fitted with time treated as a fixed effect at each step. Also, since calendar time is hypothesized to be associated with both the exposure (intervention) to the intervention and the outcome, we will adjust for this as a potential confounder in the final analysis. To account for dependence between individual measurements, an additional random effect for individuals in the study will be added into the model. The estimated intra-cluster correlation and time effect from the fitted model will be reported for use in the design of future trials and to allow appreciation of any underlying confounding effects of calendar time. The period a particular cluster has been exposed to the intervention will be accounted for in the final model as an effect modifier. For the secondary binary outcomes, we intend to employ the same methods as above for analysis. However, linear mixed-effects models accounting for temporal trends will be used to assess the intervention impact on the different continuous secondary outcomes. In the case of missing data, we will capitalize on multiple imputation techniques to handle the issue of missing data in this study.

### Data safety and management

Data will be managed using Research Electronic Data Capture (REDCap; Vanderbilt University, Nashville, USA). This will be collected by well-trained research assistants using secure password-protected tablet computers with Research Electronic Data Capture (REDCap; Vanderbilt University, Nashville, USA). All paper case report forms or source documents will be filed and stored in lockable designated cabinets and rooms. To protect participants’ confidentiality all data will be identified by anonymized study numbers. Only authorized study staff, e.g., those that collect data in the clinics will have the possibility to link the anonymized information to other participant identifiers. The data will be stored in password-protected database with no participants’ identifying information. Designated study team members will have exclusive rights to the database. Database access for other members of the study team will be determined by their responsibilities on the study, e.g., data entry, query resolution, and data monitoring. For this study, we will capitalize on single data entry. Majority of the range and consistency checks will be inbuilt into the REDCap software. In addition, the study data manager will run routine consistency checks on the study data using STATA version 15 software. At publication of the findings of this study, no identifying information will be used.

ACCESS will serve as the study coordinating center and be responsible for training, regulatory oversight, data management, and community engagement. The coordinating center will be responsible for daily operations including data collection, the training of field workers and community health workers, and participant enrollment. The study team at the coordinating center will be comprised of the project manager, the study biostatistician, and the study physician. The trial steering committee will meet every 2 weeks. This committee is comprised of the principal investigator, project manager, co-investigators, and study statistician. This team will review enrolment and study endpoints. In addition, a stakeholder and public group will be part of this study. This will comprise a community advisory board of community leaders, health works, and Ministry of Health officials. The community advisory board will meet quarterly. The PI will present the study updates as well as obtain input from the community advisory board regarding community concerns.

The composition of data monitoring committee (DMC) is as follows: we intend to have a DMC comprising of an experienced clinician, statistician, and epidemiologist. The DMC will help to ensure that the procedures are in place to ensure efficient reporting for later analysis (data processing issues to ensure the quality and efficient reporting of the data, and check compliance with the protocol by those involved in the study). Therefore, we intend to have the first meeting of the DMC at 4 months from the study start date and another meeting at 8 months post study start.

### Data monitoring

We will conduct an interim analysis at 12 months to assess differences between groups. If a statistically significant difference is detected, the intervention will be applied across the remaining clusters. We do not have any pre-defined termination rules as this is a pragmatic study with minimal risks. As such, we do not have a data and safety monitoring (DSMB) or formal termination rules as determined by the local institutional review board (IRB). Should a participant have any complaints, these will be reported to the DMC.

### Risks

This is a minimal risk study. The only intervention provided is education delivered by CHWs. As this is a pragmatic trial, the only adverse events anticipated are limited but not exclusive to a risk of loss of confidentiality, inordinate amount of participant time spent answering questions or participating in educational activities, or infections following a finger stick glucose screening. Because of the low risk, a formal DSMB will not exist, and we do not have formal termination rules as determined by the local IRB. Any potential adverse events will be reviewed by the trial steering committee and reported to the DMC. In addition, participants may experience discomfort from measuring devices such as sphygmomanometer when blood pressure is measured. Furthermore, some participants may feel uncomfortable sharing their personal information such as medical histories. Should a participant have any complaints, these will also be reported to the DMC.

### Benefits

There is no direct monetary benefit for participants. The project will generate data for the Ugandan Ministry of Health necessary to inform policy on improving treatment outcomes among hypertensive patients.

### Conflict of interest

The study team declares that there are no conflicts of interest related to this study.

### Dissemination plan

We plan to disseminate results to the beneficiaries, Makerere University College of Health Sciences, Uganda Initiative for Integrated Management of Non-Communicable Diseases (UINCD), and the Ugandan Ministry of Health, with particular interest to the NCD directorate. Additionally, we will disseminate through quarterly reports, national and international conferences, and publication(s) in a peer reviewed journal, as well as press conferences and website displays.

## Discussion

We discuss the rationale, methods, and protocols for the CHW-HTC closed cohort stepped wedge cluster randomized trial in Uganda. The overall goal of the trial is to evaluate the effectiveness of a CHW-led intervention in blood pressure control among confirmed hypertensive patients in a rural Ugandan community. Based on our statistical power calculations, we will work with a fixed number of 21 clusters, enrolling 40 participants in each cluster. Similar studies have proven applicability across various settings, strengthening our theory-informed approach. A number of studies show that CHWs can be effective in delivering patient management strategies. A randomized controlled trial based in Nepal proves that a female community health worker intervention could effectively reduce mean systolic blood pressure in hypertensive patients [26]. A Mexican-based study indicates that a CHW-led intervention among hypertensive or diabetic patients is associated with a twofold increase in the odds of disease control [27]. Similarly, a CHW-led intervention in the USA to improve hypertension management among Filipino Americans with uncontrolled blood pressure finds that blood pressure was controlled at a significantly greater percentage among the treatment group participants (83.3%) compared to the control group (42.7 %) [21].

We will follow 11 phases of implementing the intervention, introducing it within two clusters at each step of the trial and within one cluster in the final step (Fig. 2). Clusters will be assigned randomly to intervention rollout while one CHW will be attached to each cluster. We will center our intervention on monthly pro-active household visits by trained CHWs over a study period of 1 year. A local non-government organization and a multinational research group with wide-ranging experience in conducting research in rural communities will carry out the study. The local coordination will support overcoming economic and cultural barriers to enrollment and support the establishment of community trust. This clinical trial builds upon previous research and collaborations in LMIC settings investigating the advantages of engaging CHWs in the patient-centered management of hypertension.

Currently, CHW-interventions focus on the management of infectious diseases such as malaria, tuberculosis, or HIV. In addition, few studies have evaluated the impact of CHWs in NCD management such as hypertension in rural and urban settings. While these interventions have proven successful, evidence on the health outcomes of employing CHWs in a rural Ugandan setting and in the management of NCDs is limited. Hence, findings from this study will optimize disease detection and referral in rural Uganda. Furthermore, they will provide evidence-based policy recommendations on a regional and national scale. If successful, this trial has the potential to change the way we understand CHW interventions for the management of NCDs in Uganda. This may benefit patients in countries with similar socio-economic contexts by making hypertension treatments more accessible for rural communities.

## Data Availability

The datasets analyzed during the current study and statistical code are available from the corresponding author on reasonable request, as is the full protocol.

## Abbreviations

ACCESS: African Community Centre for Social Sustainability
BMI: Body mass index
CHWs: Community health workers
COPD: Chronic obstructive pulmonary disease
DSMB: Data and safety monitoring board
FBS: Fasting blood sugar
IRB: Institutional review board
LMICs: Low- and middle-income countries
MCID: Minimally important clinical difference
MOH: Ministry of Health
NCDs: Non-communicable diseases
NIH: National Institute of Health
PI: Principal investigator
RBS: Random blood sugar
SSA: Sub-Saharan Africa
WHO: World Health Organization
WHO PEN: Package of Essential Non-Communicable Diseases Interventions
